# Self-Determination for Nurses

**Published:** 1920-06-12

**Authors:** 


### SELF-DETERMINATION FOR NURSES.

### Clap-trap Phraseology.

Phrases, like habits, are great helps, or great hindrances. We live in an era of subtle propaganda, and phrases are being bandied round the world with the object of catching the innocent, as the fowler lays his birdlime snare. During the later stages of the war, enemy countries, homes of tyranny and autocracy, clamoured for "the freedom of the seas," meaning thereby something entirely different from what the words convey. Now we have on every hand preached the doctrine of "self-determination." Self-determination, as popularly expounded, sounds well, but will not bear analysis. The trade union is supposed to be the worker's way to paradise, and Self-determination is written over the gate. But self-abnegation characterises the mental and spiritual attitude of its members. Once you enter you cease to think. You simply obey.

I am a complete believer in self-determination for the individual and the mass within the narrow limits of its possible application. These, be it observed, are strictly defined. You cannot drive your carriage down the Strand in any go-as-you-please fashion, nor by taking thought can you add one cubit to your stature. Not even of Napoleon can it be said that he was the master of his destiny. The freest of us are more or less in bondage. It is extremely important that we should think clearly, for nothing is easier than, in endeavouring to be free, to throw off one form of slavery in substitution for another and perhaps a worse form.

### Silence Approximates to Death.

The first step towards self-determination in the high sense is self-expression—articulation. What one says may be foolish or stupid. A child may want very badly to play with the match-box, or a typhoid patient may crave for meat. It is better all the same that they should say so than be silent. It is at least a sign of animate life, whereas silence approximates to death. Silent individuals or communities are unprogressive, and being unprogressive they quickly become retrogressive.

I know of no community more inarticulate than that of the British Nurses. It is probably a result of hard work and severe discipline, and although these may in themselves be good in reasonable measure, if they produce dumbness there ought to be a corrective. Leaders of the nursing profession should realise that the silence of its members does not make for progress, but rather for deterioration.

### Elections Without Intelligence.

In this connection I should like to put a question to the College of Nursing Executive, and likewise to the many thousands of its members. In all the College elections that have been held from year to year, for or against what principle or principles were the nurses' votes recorded? Can anybody hazard a guess? Quite a number of vital questions have appeared on the screen, such as nurses' hours, nurses' pay, nurses' pensions, the Nation's Tribute to Nurses, and, not least, the position of the nurse in Health Reconstruction. Have the nurses who are College members ever nominated a candidate to champion any one of these? Or, can any College member form an intelligent opinion as to the active or passive attitude of the Executive concerning these? Is there, or has there ever been published, any document which would enable a seeker after truth to find it? I must confess that I am able to find only a blank page.

Let it not be supposed that I make any such suggestion as that the College is inactive, or neglectful of the nurses' interests. Now and then there is a sunburst which proves the opposite, as, for example, the fifty-six hours maximum recently suggested in connection with the Hours of Employment Bill, which, if not a guarantee of ideal conditions, will, if made law, prevent sweating.

This is excellent, and all credit to the College for its well-conceived scheme. This, however, does not touch my argument, which is an argument against a policy of silence.

### The Case Illustrated.

Let me illustrate. The Minister of Health has, with the co-operation of the Board of Education, granted a monopoly to university institutions to prepare recruits for the position of health visitor in England. A certain concession, which reads well enough on paper, has been granted to the trained nurse. She need not spend so much time at her preparation in such an institution as an outsider. But it is obvious that for practical purposes the concession is of no value. It is physically impossible for the great mass of nurses to surrender their employment and take up their abode for twelve months near a university centre. In effect, the Minister of Health has arbitrarily closed the door to the trained nurse.

Now neither the Minister of Health nor the Board of Education are private employers. They represent the people of England, and the people of England it is who will pay. I venture to challenge the right of the Minister of Health to prescribe any school of training for State servants unless he is prepared to defray the cost of the training there given. He has not only a right but a duty to see that recruits are properly qualified, and therefore an examination scheme was to be expected. London University, since its foundation, granted degrees to private students, and by this means gave immense stimulus to education. London University is not a State department, and I maintain that the State doors should be open wide so that every citizen should have at least a chance of entering. Let the Minister of Health and the Board of Education set out their requirements as to knowledge, experience, and general fitness. The fixing of such a standard is the limit of their legitimate functions.

### An Absence of Articulation.

This is a very large and important question. But I cannot find that it is one concerning which there is any public opinion either in the College Executive or amongst the College members. There is an absence of articulation—silence, in fact. I mention this, as I have stated, as an illustration. Was it considered at the recent election either by the nominators or the nominees? If it had been, pressure could immediately be brought to bear in Parliament. Unless such pressure is exercised the Ministers responsible are quite justified in assuming that their attitude is generally approved.

IERNE.

